# Re-routing of Sugar Catabolism Provides a Better Insight Into Fungal Flexibility in Using Plant Biomass-Derived Monomers as Substrates

**DOI:** 10.3389/fbioe.2021.644216

**Published:** 2021-03-08

**Authors:** Tania Chroumpi, Mao Peng, Lye Meng Markillie, Hugh D. Mitchell, Carrie D. Nicora, Chelsea M. Hutchinson, Vanessa Paurus, Nikola Tolic, Chaevien S. Clendinen, Galya Orr, Scott E. Baker, Miia R. Mäkelä, Ronald P. de Vries

**Affiliations:** ^1^Fungal Physiology, Westerdijk Fungal Biodiversity Institute & Fungal Molecular Physiology, Utrecht University, Utrecht, Netherlands; ^2^Environmental Molecular Science Laboratory, Pacific Northwest National Laboratory, Richland, WA, United States; ^3^Department of Microbiology, University of Helsinki, Helsinki, Finland

**Keywords:** lignocellulosic substrates, pentose catabolic pathway, D-galacturonic acid catabolic pathway, L-rhamnose catabolic pathway, wheat bran, sugar beet pulp, CAZymes, *Aspergillus niger*

## Abstract

The filamentous ascomycete *Aspergillus niger* has received increasing interest as a cell factory, being able to efficiently degrade plant cell wall polysaccharides as well as having an extensive metabolism to convert the released monosaccharides into value added compounds. The pentoses D-xylose and L-arabinose are the most abundant monosaccharides in plant biomass after the hexose D-glucose, being major constituents of xylan, pectin and xyloglucan. In this study, the influence of selected pentose catabolic pathway (PCP) deletion strains on growth on plant biomass and re-routing of sugar catabolism was addressed to gain a better understanding of the flexibility of this fungus in using plant biomass-derived monomers. The transcriptome, metabolome and proteome response of three PCP mutant strains, Δ*larA*Δ*xyrA*Δ*xyrB*, Δ*ladA*Δ*xdhA*Δ*sdhA* and Δ*xkiA*, grown on wheat bran (WB) and sugar beet pulp (SBP), was evaluated. Our results showed that despite the absolute impact of these PCP mutations on pure pentose sugars, they are not as critical for growth of *A. niger* on more complex biomass substrates, such as WB and SBP. However, significant phenotypic variation was observed between the two biomass substrates, but also between the different PCP mutants. This shows that the high sugar heterogeneity of these substrates in combination with the high complexity and adaptability of the fungal sugar metabolism allow for activation of alternative strategies to support growth.

## Introduction

The majority of industrial processes for the production of chemicals, materials, and energy are still based on fossil fuels, especially coal and crude oil. However, to gain independence from these raw materials, more consideration has been given in the last decades to the use of renewable materials and agricultural residues as promising low-cost feedstocks for obtaining high added-value products.

The filamentous fungus *Aspergillus niger* is one of the most prominent fungal cell factories used in biotechnology. It is known for its ability to naturally degrade complex plant biomass polysaccharides, including both cell wall (cellulose, hemicellulose and pectin) and storage (inulin and starch) components, into simple sugars using a rich arsenal of Carbohydrate-Active Enzymes (CAZymes) (de Vries and Visser, 2001; Lombard et al., 2014; Benoit et al., 2015). Despite the complexity of the polysaccharides forming the cell wall, their backbone is mainly formed by simple sugars, such as D-glucose, D-xylose, L-arabinose, D-galactose, D-galacturonic acid, D-fructose and L-rhamnose (Somerville, 2006; Mohnen, 2008; Scheller and Ulvskov, 2010; Ochoa-Villarreal et al., 2012). In nature, fungi need to first recognize the plant biomass components to produce the right set of CAZymes that can break down the complex structures into these simple molecules. The resulting sugars are subsequently transported into the cell and converted into energy and intermediate metabolites through a wide range of metabolic pathways (Khosravi et al., 2015). An in-depth understanding of the *A. niger* metabolic network will provide a detailed blueprint for the metabolic engineering of this fungus to improve productivity of a broad range of proteins and metabolites.

**FIGURE 1:**
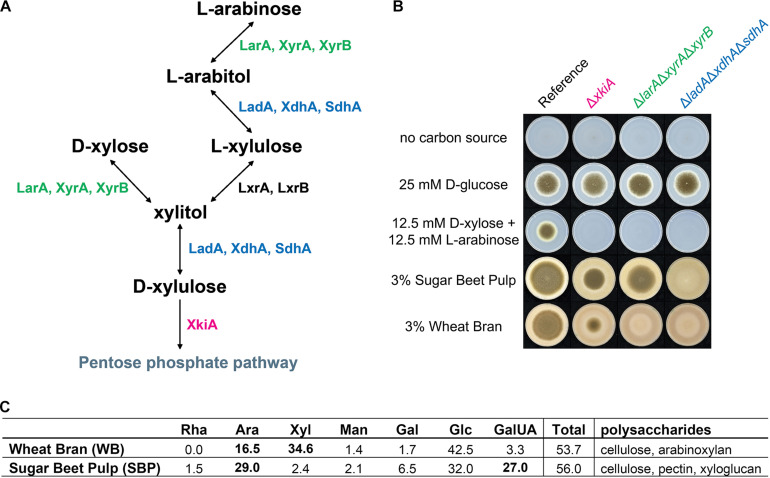
**(A)** Pentose catabolic pathway (PCP) in *Aspergillus niger*. LarA = L-arabinose reductase, LadA = L-arabitol dehydrogenase, LxrA and LxrB = L-xylulose reductases, SdhA = sorbitol dehydrogenase, XyrA and XyrB = D-xylose reductases, XdhA = xylitol dehydrogenase, XkiA = D-xylulose kinase. **(B)** Growth profile of the *A. niger* reference strain (N593 ΔkusA) and the PCP deletion mutants on solid MM with or without addition of carbon source. Strains were grown for 5 days at 30°C and **(C)** composition analysis of polymeric carbon sources, wheat bran (WB) and sugar beet pulp (SBP), used for growth profile and multi-omics analysis of PCP mutants. Rha = rhamnose, Ara = arabinose, Xyl = xylose, Man = mannose, Gal = galactose, Glc = glucose, GalUA = galacturonic acid. Concentration in mol%.

The pentoses L-arabinose and D-xylose are the most abundant monosaccharides in nature after D-glucose, being major components of the hemicelluloses xylan and xyloglucan, and of pectin (Seiboth and Metz, 2011). In most fungi, L-arabinose and D-xylose are metabolized through the pentose catabolic pathway (PCP) (Witteveen et al., 1989), through oxidation, reduction and phosphorylation reactions to finally form D-xylulose-5-phosphate, which enters the pentose phosphate pathway (PPP) (Seiboth and Metz, 2011; Figure 1A). Although pentose catabolism is among the best studied pathways of *A. niger* primary carbon metabolism, the simplistic view of this pathway has recently been challenged (Chroumpi et al., 2021). Due to the residual growth of the PCP single deletion mutants, identification of additional genes involved in pentose catabolism was achieved: a second D-xylose reductase (XyrB), a second L-xylulose reductase (LxrB) and the role of sorbitol dehydrogenase (SdhA) in compensating for the loss of L-arabitol dehydrogenase (LadA) and xylitol dehydrogenase (XdhA) (Figure 1A). Additionally, all enzymatic steps of the PCP in *A. niger*, apart from the last one, were shown to be catalyzed by multiple enzymes, which together ensure efficient conversion of pentose sugars.

In this study, the influence of selected PCP deletion strains on growth on plant biomass and re-routing of sugar catabolism was analyzed to gain a better understanding of the flexibility of this fungus in using plant biomass-derived monomers as substrates. The transcriptome, metabolome and proteome responses of three pentose catabolic mutant strains, Δ*larA*Δ*xyrA*Δ*xyrB*, Δ*ladA*Δ*xdhA*Δ*sdhA* and Δ*xkiA*, grown on two plant biomass substrates, wheat bran (WB) and sugar beet pulp (SBP), were evaluated. These mutants have been previously shown to block pentose catabolism at different steps of the pathway and as a result accumulate different intermediates that could act as inducers (Chroumpi et al., 2021). While both substrates contain cellulose, WB is rich in arabinoxylan and SBP contains xyloglucan and pectin (Figure 1C). This means that both substrates contain considerable amounts of L-arabinose and D-xylose, making them highly suitable for analysis of this pathway.

## Materials and Methods

### Strains, Media, and Growth Conditions

The *A. niger* strains used in this study are listed in Table 1. The strains were grown at 30°C using Minimal Medium (MM, pH 6) or Complete Medium (CM, pH 6) with the appropriate carbon source (de Vries et al., 2004). For solid cultivation, 1.5% (w/v) agar was added in the medium and, unless stated otherwise, all agar plates contained 1% D-glucose as carbon source. When required, media of auxotrophic strains were supplemented with 1.22 g/L uridine.

**TABLE 1:** *A. niger* strains used in this study.

Strains	Gene ID	Enzyme activity	CBS number	Genotype	References
Reference strain (N593 Δ*kusA*)	–	–	CBS 138852	*A. niger* N593, *cspA1*, *kusA*:*amdS*, *pyrG*^–^	Meyer et al., 2007
Δ*larA*Δ*xyrA*Δ*xyrB*	NRRL3_10050 (*larA*) NRRL3_01952 (*xyrA*) NRRL3_10868 (*xyrB*)	L-arabinose/ D-xylose reductase	CBS 144530	*A. niger* N593, *cspA1*, *kusA*:*amdS*, *pyrA*^–^, ***larA^–^, xyrA^–^, xyrB^–^***	Chroumpi et al., 2021
Δ*ladA*Δ*xdhA*Δ*sdhA*	NRRL3_02523 (*ladA*) NRRL3_09204 (*xdhA*) NRRL3_04328 (*sdhA*)	L-arabitol/xylitol dehydrogenase	CBS 144672	*A. niger* N593, *cspA1*, *kusA*:*amdS*, *pyrA*^–^, ***ladA^–^, xdhA^–^, sdhA^–^***	Chroumpi et al., 2021
Δ*xkiA*	NRRL3_04471 (*xkiA*)	xylulokinase	CBS 144042	*A. niger* N593, *cspA1*, *kusA*:*amdS*, *pyrA*^–^, ***xkiA*^–^**	Chroumpi et al., 2021

For growth profiling, 6 cm petri dishes with vents containing MM agar supplemented with 25 mM D-glucose (Sigma, G8270), a mixture of 12.5 mM D-xylose (Sigma, 95729) and 12.5 mM L-arabinose (Sigma, A3256), 3% wheat bran (WB) or 3% sugar beet pulp (SBP) were used. The monosaccharide composition analysis of WB and SBP is presented in Figure 1C. Spores were harvested from CM agar plates in ACES buffer, after five days of growth, and counted using a hemocytometer. Growth profiling plates were inoculated with 1,000 spores in 2 μl, and incubated at 30°C for 5 days.

All liquid cultures were incubated in an orbital shaker at 250 rpm and 30°C. For transfer experiments, the pre-cultures containing 250 mL CM with 2% D-fructose in 1 L Erlenmeyer flasks were inoculated with 10^6^ spores/mL and incubated for 16 h. Subsequently, the mycelia were harvested by filtration on sterile cheesecloth, washed with MM and ∼0.5 g (dry weight) was transferred to 250 mL Erlenmeyer flasks containing 50 mL MM supplemented with 1% WB or 1% SBP. All cultures were performed in biological triplicate as were all the subsequent analyses. After 2, 8, and 24 h of incubation, the mycelia were harvested by vacuum filtration, dried between tissue paper and frozen in liquid nitrogen. Culture filtrates were also harvested for extracellular metabolomics and proteomics analysis. All samples were stored at −80°C until being processed.

### Transcriptome Sequencing and Analysis

The transcriptomic response of the reference strain and the PCP deletion mutants induced after 2, 8, and 24 h on 1% WB or 1% SBP was analyzed using RNA-seq analysis. Total RNA was extracted from ground mycelial samples using TRIzol^®^ reagent (Invitrogen) and purified with the NucleoSpin^®^ RNA Clean-up Kit (Macherey-Nagel), while contaminant gDNA was removed by rDNase treatment directly on the silica membrane. The RNA quality and quantity were analyzed with a RNA6000 Nano Assay using the Agilent 2100 Bioanalyzer (Agilent Technologies). Purification of mRNA, synthesis of cDNA library and sequencing were conducted at the Environmental Molecular Sciences Laboratory (EMSL).

RNA samples were assessed using the Agilent 2100 Bioanalyzer. The TruSeq stranded mRNA (cat#20020594) was used to generate cDNA library for illumina NextSeq550 platform according to the manufacture protocol. Single-read sequencing of the cDNA libraries with a read length of 150 was performed with NextSeq 500 Sequencing System using NextSeq 500/550 High Output v2 kit 150 cycles (cat#20024907). Data quality was assessed using FastQC^1^. Reads were aligned to the *A. niger* NRRL 3 genome (Aguilar-Pontes et al., 2018) using bowtie2^2^, with parameters -local, –sensitive-local. The RNAseq data set was deposited at the GEO (Barrett et al., 2012) database under the accession number GSE162901. Reads were aligned to genes using HTSeq−count (Anders et al., 2014) with parameters -a = 1, –mode = “union”. The analysis was performed on three independent biological replicates. Differential gene expression was assessed using the R package DESeq2 (Love et al., 2014), with all subsequent analysis performed in R unless otherwise stated. Transcripts were considered differentially expressed if the DESeq2 fold change was >2 or <0.5 and Padj < 0.01. Transcripts with FPKM ≤ 50 were considered lowly (i.e., not substantially) expressed. MDS plots were also generated using DESeq2.

The Gene Ontology (GO) annotation was retrieved from JGI MycoCosm database^3^ and the Gene Ontology (GO) annotation database from R Bioconductor was used to map their ancestor nodes in the GO hierarchy. The GO Slim terms defined in AspGD^4^ were selected for enrichment analysis. The GO biological process terms enriched within the significant differentially expressed gene lists compared to the genome background were detected by a hypergeometric distribution model calculated with in-house script. The *P*-values for multiple tests were corrected with Benjamini and Hochberg’s method, and significantly enriched GO terms were selected with *P*-values <0.01.

### Proteomics Data Generation and Analysis

Equivalent volumes of culture supernatant were extracted using the MPLEx protocol (Nakayasu et al., 2016). The protein interlayer from the extraction was then resuspended in an 8 M urea solution, reduced with DTT, digested with Trypsin, put through C18 SPE for clean-up, and diluted to 0.1 μg μL^–1^ for LC-MS/MS.

MS analysis was performed using a Q−Exactive Plus mass spectrometer (Thermo Scientific) outfitted with a homemade nano−electrospray ionization interface. Electrospray emitters were homemade using 150 μm o.d. × 20 μm i.d. chemically etched fused silica (Kelly et al., 2006). The ion transfer tube temperature and spray voltage were 250°C and 2.2 kV, respectively. Data were collected for 120 min following a 10 min delay after completion of sample trapping and start of gradient. FT−MS spectra were acquired from 300 to 1,800 m/z at a resolution of 30 k (AGC target 3e6) and while the top 12 FT−HCD−MS/MS spectra were acquired in data−dependent mode with an isolation window of 1.5 m/z and at a resolution of 17.5 k (AGC target 1e5) using a normalized collision energy of 30 s exclusion time.

Generated MS/MS spectra were searched using the mass spectral generating function (MSGF +) algorithm (Kim et al., 2008; Kim and Pevzner, 2014) against the *A. niger* translated genome sequence available from Aspni_NRRL3_1 (Aguilar-Pontes et al., 2018). MSGF + was used in target/decoy mode with 20 ppm parent ion tolerance, partial tryptic rule and methionine oxidation (+ 15.9949) as dynamic modification. Best matches from the MSGF + searches were filtered at 1% FDR and only protein specific peptides were used in consequent aggregation and quantitative analysis. Relative peptide abundances can be determined by calculating the area under the curve of the peptide ion peak in the MS measurement. This was accomplished using MASIC software (Monroe et al., 2008)^5^ and results were aggregated using MS SQL (Microsoft) database. InfernoRDN software (Polpitiya et al., 2008)^6^ was used to transform peptides abundances (log2) and perform mean central tendency normalization. Protein grouped normalized peptide abundances were de-logged, summed, transformed (log2) and normalized again in InfernoRDN to produce normalized abundances for the protein level roll-up. For an identified protein to be considered differentially produced, the requirements were a fold change of the mean intensity values of >2 or <0.5 and Padj < 0.05 from a two−tailed *t* −test of the log2 transformed intensity values. Note that where an intensity value was not detected for a protein in a sample, a zero value was used. The mass spectrometry proteomics data have been deposited to the ProteomeXchange Consortium via the MassIVE partner repository with the data set identifier (PXD023205).

### Metabolomics Data Generation and Analysis

Dried metabolite extracts from samples were derivatized using a modified version of the protocol used to create FiehnLib (Fiehn, 2016). Samples underwent methoximation to protect carbonyl groups and reduce tautomeric isomers, followed by silylation with *N*-Methyl-*N*-(trimethylsilyl) trifluoroacetamide and 1% trimethylchlorosilane (MSTFA) to derivatize hydroxy and amine groups to trimethylsilated (TMS) forms. GC/MS data were collected over a mass range of 50–550 m/z using an Agilent GC 7890A coupled with a single quadrupole MSD 5975C (Agilent Technologies). A standard mixture of fatty acid methyl esters (FAMEs) (C8-C28) was analyzed with samples for RI alignment. The GC oven was held at 60°C for 1 min after injection, followed by a temperature increase of 10°C min^–1^ to a maximum of 325°C at which point it was held for 5 min.

Agilent.D files were converted to netCDF format using Agilent Chemstation. GC-MS raw data files were converted to binary files and processed using MetaboliteDetector software (version 2.5 beta) (Hiller et al., 2009). Retention indices (RIs) of detected metabolites were calculated based on analysis of the Fatty acid Methyl Esters standard mixture followed by chromatographic deconvolution and alignment. Metabolites were initially identified by matching experimental spectra to an augmented version of FiehnLib (Kind et al., 2009). All metabolite identifications were manually validated with the NIST 14 GC–MS library. The summed abundances of the three most abundant fragment ions of each identified metabolite were integrated across the GC elution profile (automatically determined by MetaboliteDetector). Fragment ions due to trimethylsilylation (that is, m/z 73 and 147) were excluded from the determination of metabolite abundance. Features resulting from GC column bleeding were removed from the data before further data processing and analysis.

## Results

### The Different *A. niger* PCP Deletion Mutants Cause Significant Phenotypic Variation on Lignocellulosic Biomass Substrates

Selected PCP gene deletion mutants that block conversion of both pentoses at different pathway steps (Figure 1A), and thus result in accumulation of different PCP intermediates, were grown on a mixture of the monosaccharides L-arabinose and D-xylose, and on the biomass substrates WB and SBP (Figure 1B). As expected, all three Δ*larA*Δ*xyrA*Δ*xyrB*, Δ*ladA*Δ*xdhA*Δ*sdhA* and Δ*xkiA* mutants were unable to grow on the pentose mixture, while these deletions resulted in reduced growth on WB and SBP, compared to the reference strain (Figure 1B). The extent of the growth reduction depended on the mutant strain and the substrate. The Δ*larA*Δ*xyrA*Δ*xyrB* mutant, which blocks the first step of pentose conversion, was practically unable to grow on WB, but showed only a small growth reduction on SBP. In contrast, growth of Δ*ladA*Δ*xdhA*Δ*sdhA* was similarly affected as that of the triple reductase mutant on WB, but was nearly abolished on SBP. Finally, the growth of Δ*xkiA* mutant, was reduced compared to the reference strain, but not abolished on both tested biomass substrates.

The rescued growth of Δ*xkiA* mutant on both biomass substates and of Δ*larA*Δ*xyrA*Δ*xyrB* mutant on SBP (Figure 1B), could suggest the expression of genes encoding alternative kinases and reductases, respectively, with sufficient specificity for the accumulated PCP intermediates to support growth. Following these observations, the re-routing of sugar metabolism in order to support growth of these PCP mutants on WB and SBP was further analyzed by multi-omics analysis. Mycelia of the reference strain and the PCP mutants were transferred to WB and SBP, and both mycelial and supernatant samples were harvested after 2, 8 and 24 h.

### The PCP Deletions Affect the Transcriptome Abundance of Metabolic and CAZy Genes on WB and SBP

**FIGURE 2:**
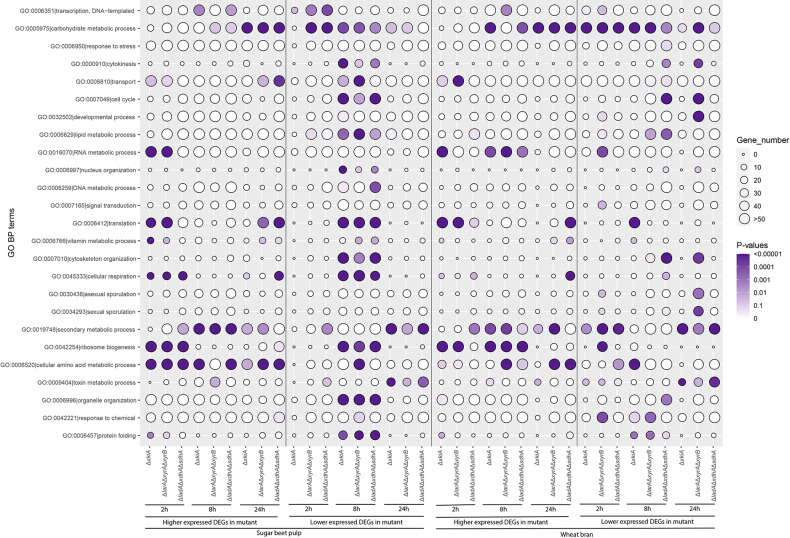
Gene Ontology (GO) terms associated with the function of genes upregulated or downregulated during growth on sugar beet pulp (SBP) and wheat bran (WB) in the *A. niger* reference strain (N593 Δ*kusA*) and the PCP deletion mutants. The size and color of the circles represent the number of genes and statistical significance of enriched GO terms, respectively.

GO enrichment analysis of the expression data of the PCP mutants revealed a significant effect of these mutations on both primary and secondary metabolic responses of the fungus (Figure 2). During growth on both substrates, the expression of genes particularly involved in carbohydrate metabolic processes (GO:0005975), but also in cellular amino acid metabolic processes (GO:0006520) and in ribosome biogenesis (GO:0042254) was elevated compared to the reference strain. However, transport processes (GO:0006810) and metabolic processes do not seem to be synchronized. This is in line with the results of a previous study (Mäkelä et al., 2018), where sugar transport and metabolism were shown not to be co-regulated during growth of *A. niger* in liquid cultures. Interestingly, after 8 h of growth on SBP, an overall repression of genes involved in most of the studied GO terms was observed for all three mutants.

In this study, in order to investigate the adaptation strategy of each PCP mutant to these biomass substrates, we mainly focused on the genes encoding carbon catabolic enzymes and CAZymes involved in plant biomass degradation.

### The Upregulation of the D-Galacturonic Acid and L-Rhamnose Catabolic Pathway Genes Could Partly Explain the Rescued Growth of the Δ*larA*Δ*xyrA*Δ*xyrB* and Δ*xkiA* Mutants on SBP

**FIGURE 3:**
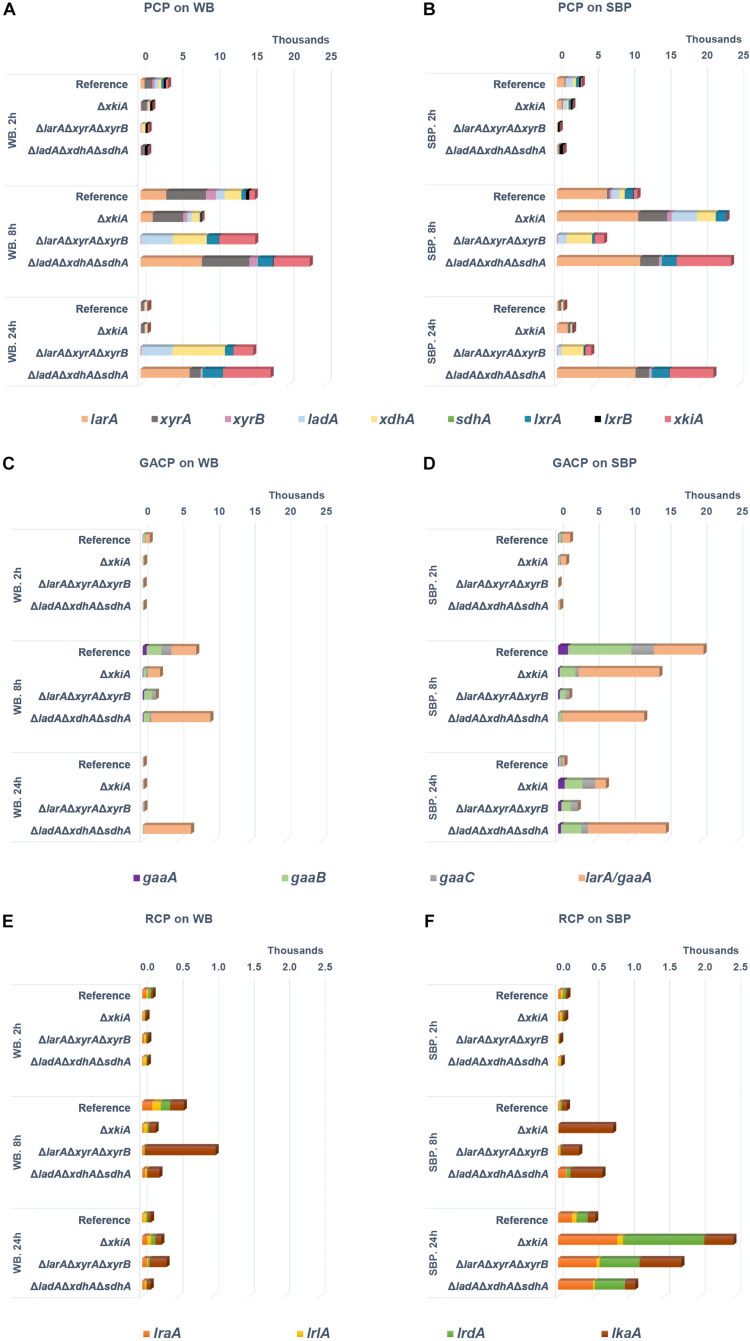
Expression levels (FPKM) of catabolic genes during growth on **(A,C,E)** wheat bran (WB) and **(B,D,F)** sugar beet pulp (SBP) in the *A. niger* reference strain (N593 Δ*kusA*) and the PCP deletion mutants. PCP, pentose catabolic pathway; GACP, D-galacturonic acid catabolic pathway; RCP, L-rhamnose catabolic pathway. Genes encoding L-arabinose reductase (*larA*), L-arabitol dehydrogenase (*ladA*), L-xylulose reductases (*lxrA* and *lxrB*), sorbitol dehydrogenase (*sdhA*), D-xylose reductases (*xyrA* and *xyrB*), xylitol dehydrogenase (*xdhA*), D-xylulose kinase (*xkiA*), D-galacturonic acid reductase (*gaaA*), L-galactonate dehydratase (*gaaB*), 2-keto-3-deoxy- L-galactonate aldolase (*gaaC*), L-glyceraldehyde reductase (*larA*/*gaaD*), L-rhamnose-1-dehydrogenase (*lraA*), L-rhamnono-γ-lactonase (*lrlA*), L-rhamnonate dehydratase (*lrdA*), L-2-keto-3-deoxyrhamnonate aldolase (*lkaA*).

**FIGURE 4:**
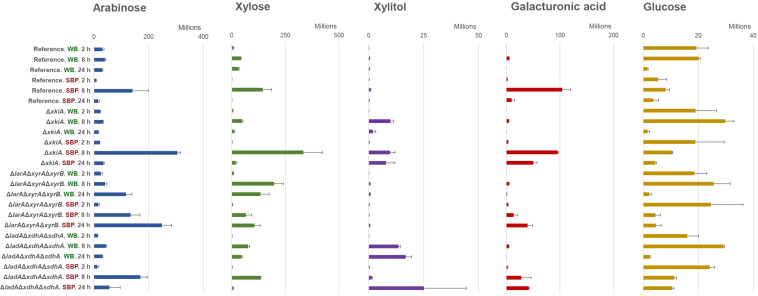
Metabolite abundance in culture supernatant of arabinose, xylose, xylitol, galacturonic acid and glucose, after 2, 8, and 24 h transfer of the *A. niger* reference strain (N593 Δ*kusA*) and the PCP deletion mutants to 1% (w/v) wheat bran (WB) and 1% (w/v) sugar beet pulp (SBP). The error bars represent the standard deviation between three biological replicates.

Deletion of the PCP genes in the mutant strains led to altered expression of the remaining PCP genes on WB and SBP. On both biomass substrates, the expression of the remaining PCP genes increased after 8 h in all strains (Figures 3A,B and Supplementary Table 1). This is probably due to the accumulation of pentoses and polyols that have been previously indicated as potential inducers of the AraR and XlnR transcriptional activators of the PCP genes (de Vries, 2003; de Groot et al., 2007; Battaglia et al., 2011a, b). However, their expression in the reference and the Δ*xkiA* strains strongly reduced after 24 h on both biomass substrates, while they remained at significantly high levels in Δ*larA*Δ*xyrA*Δ*xyrB* and Δ*ladA*Δ*xdhA*Δ*sdhA* (Figures 3A,B). Apart from the reference strain, the Δ*xkiA* mutant was the only strain that was still able to grow on both WB and SBP (Figure 1B). This indicates that the depletion of the pentose sugars and pathway intermediates under these conditions might be the reason of the observed reduction in expression of the PCP genes in these strains (Figure 4). The depletion of the pentose sugars in the Δ*xkiA* mutant could be explained by the presence of enzymes with some kinase activity on D-xylulose, of which the corresponding genes are induced under these conditions.

Similar to the PCP genes, the expression of genes involved in other carbon metabolic pathways was also affected. In particular, the expression of the genes involved in the catabolism of D-galacturonic acid was also significantly upregulated in the PCP deletion mutants compared to the reference strain on SBP (Figure 3D and Supplementary Table 1). On WB, which does not contain substantial amounts of D-galacturonic acid (Figure 1C), expression of the D-galacturonic acid catabolic pathway (GACP) genes was not induced (Figure 3C and Supplementary Table 1). Only the expression of *larA/gaaD*, encoding the enzyme involved in the last step of the GACP, was strongly induced on both substrates, since it is the same enzyme involved in the first step of L-arabinose metabolism in *A. niger* (Martens-Uzunova and Schaap, 2008; Mojzita et al., 2010). The expression of this gene was higher in the Δ*ladA*Δ*xdhA*Δ*sdhA* mutant on both substrates (Figures 3C,D and Supplementary Table 1) showing that its induction is mainly a result of L-arabitol accumulation in this strain.

Finally, the expression of the genes involved in L-rhamnose catabolism was also upregulated on SBP (Figure 3E,F and Supplementary Table 1), while the absence of L-rhamnose in WB (Figure 1C) resulted in no expression of the L-rhamnose catabolic pathway (RCP) genes on this substrate (Figure 3C). In contrast to the other pathway genes, significant upregulation of *lkaA*, which was previously shown to be involved in the last step of L-rhamnose catabolism (Chroumpi et al., 2021), was observed on both substrates after 8 h of incubation.

Interestingly, albeit both biomass substrates are rich in D-glucose, increased expression of the glycolytic genes was not observed for any of the PCP mutants (data not shown).

### The Rescued Growth of the Δ*larA*Δ*xyrA*Δ*xyrB* and Δ*xkiA* Mutants on SBP Relies in Activation of Different Carbon Catabolic Re-routing Strategies

Similar to recently results on pure pentose sugars (Chroumpi et al., 2021), no significant accumulation of arabinose, xylose or other PCP intermediates were observed during growth on WB and SBP in the reference strain (Figure 4). Since the PCP remains intact in this strain, the released pentose sugars can efficiently be catabolized and used to support growth.

In the Δ*larA*Δ*xyrA*Δ*xyrB* mutant, accumulation of arabinose and xylose occurred after 8 and 24 h of growth on SBP and WB (Figure 4). Although the Δ*larA*Δ*xyrA*Δ*xyrB* mutant could grow on SBP, the amount of accumulated pentose sugars increased with time. This observation supports our previous conclusion that this mutant cannot utilize the pentose sugars for growth. The absence of polyol accumulation also suggests that under these conditions no alternative enzymes are induced which are able to convert arabinose and xylose into their respective polyols. As expected, accumulation of D-galacturonic acid was also observed in the Δ*larA*Δ*xyrA*Δ*xyrB* mutant at the later time points on SBP (Figure 4), due to the fact that the last step of the pathway is also disrupted after deletion of *larA*/*gaaD*. However, the growth of this mutant on SBP indicates the activation of other catabolic pathways that allow its adaptation under these conditions. Interestingly, glucose was shown to be significantly reduced after 8 and 24 h on SBP (Figure 4), indicating that it might be used as an alternative carbon source to support growth of the Δ*larA*Δ*xyrA*Δ*xyrB* mutant.

In the Δ*xkiA* mutant, which was able to grow on both biomass substrates (Figure 1B), the accumulated arabinose and xylose observed after 8 h of growth on SBP and WB were depleted after 24 h (Figure 4). In this mutant, a similar consumption pattern was also observed for the accumulated arabitol and xylitol. These observations again support our previous hypothesis for the presence of alternative enzymes induced under these conditions, which may facilitate the conversion of pentose sugars and of PCP intermediates in *A. niger*. The limited presence of the PCP inducers can also justify the reduced expression of the PCP genes after 24 h on both substrates (Figures 3A,B).

Finally, in the Δ*ladA*Δ*xdhA*Δ*sdhA* mutant, the accumulated arabinose and xylose measured after 8 h of growth on SBP and WB were also depleted after 24 h (Figure 4). However, in contrast to the Δ*xkiA* mutant, significant accumulation of their respective polyols followed the depletion of the pentose sugars, showing that this was not a result of their use to support growth but their conversion into further downstream PCP intermediates. In this mutant, accumulation of D-galacturonic acid and glucose was also observed at the later time points on SBP, suggesting that neither of these sugars could be used as alternative carbon sources.

### Significant Variation in Expression of the CAZy Genes Involved in Utilization of Arabinoxylan, Cellulose and Xyloglucan Was Observed Between the PCP Mutants

**FIGURE 5:**
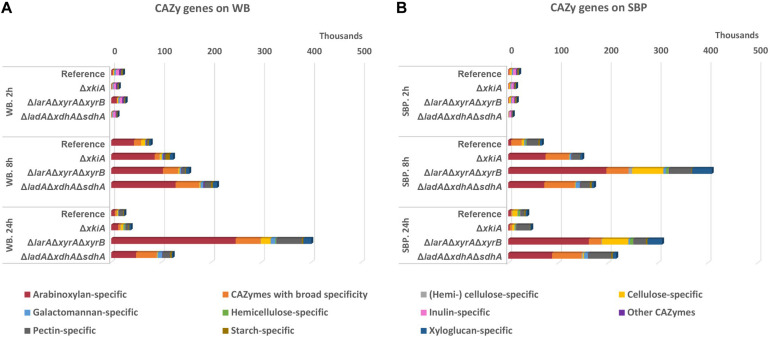
Expression levels (FPKM) of genes encoding CAZymes during growth on **(A)** wheat bran (WB) and **(B)** sugar beet pulp (SBP) in the *A. niger* reference strain (N593 Δ*kusA*) and the PCP deletion mutants.

Expression of Carbohydrate Active Enzymes (CAZymes) was also impacted in the PCP deletion mutants (Figure 5 and Supplementary Table 2) on both biomass substrates. Based on their substrate specificity, these enzymes were divided into 11 different sub-groups (Figure 5 and Supplementary Table 2). The substrate specific enzyme sub-groups that showed the highest differences in the mutants compared to the reference strains were the ones involved in degradation of the polysaccharides arabinoxylan, cellulose and xyloglucan, justified by the composition of WB and SBP (Figure 1C). The arabinoxylan-specific sub-group comprised of several genes encoding β-1,4 endoxylanases (XLN), β-1,4 xylosidases (BXL), arabinoxylan arabinofuranohydrolase (AXH), α-glucoronidase (AGU) and acetyl xylan esterases (AXE). The cellulose−specific sub−group included genes encoding β-1,4-glucosidases (BGL), β−1,4−endoglucanases (EGL), cellobiohydrolases (CBH) and cellobiose dehydrogenases (CDH), while the xyloglucan−specific sub−group included xyloglucanases (XG-EGL), α−xylosidases (AXL) and α−fucosidases (AFC).

In *A. niger*, the transcriptional activator XlnR and AraR have been shown to regulate the expression of (hemi-)cellulolytic genes (van Peij et al., 1998a; de Vries, 2003), and arabinanolytic genes (Battaglia et al., 2011b), respectively. Genes under control of XlnR encode arabinoxylan−active enzymes, such as β-1,4 endoxylanases (*xlnB*, *xlnC*) (van Peij et al., 1998a, b), β-1,4 xylosidase (*xlnD*) (van Peij et al., 1998a, b), α−glucuronidase (*aguA*) (van Peij et al., 1998b; de Vries et al., 2002), acetyl xylan esterase (*axeA*) (van Peij et al., 1998b) and arabinoxylan arabinofuranohydrolase (*axhA*) (van Peij et al., 1998b), but also cellulose−active enzymes, such as β−endoglucanases (*eglA*, *eglB*, *eglC*) (van Peij et al., 1998b) and cellobiohydrolases (*cbhA*, *cbhB*) (Gielkens et al., 1999), and CAZymes with broad specificity such as feruloyl esterase (*faeA*) (van Peij et al., 1998b), α−galactosidase (*aglB*) (de Vries et al., 1999a) and β−galactosidase (*lacA*) (de Vries et al., 1999a). The transcriptional regulator AraR controls the expression of genes encoding α−arabinofuranosidases (*abfA*, *abfB*) in *A. niger* (Battaglia et al., 2011b).

The expression of the arabinoxylan-specific gene sub-group on both substrates remained low in the reference strain, where the accumulation of the XlnR and AraR inducers has been shown to remain at significantly lower levels compared to the PCP mutants (Chroumpi et al., 2021). In general, growth on WB, which is a particularly rich in arabinoxylan, led to higher expression of the genes encoding arabinoxylan-specific enzymes compared to the other groups of enzymes (Figure 5A and Supplementary Table 2). After 24 h of growth on WB, an even stronger upregulation of these genes was observed, coinciding with higher accumulation of pentoses in the Δ*larA*Δ*xyrA*Δ*xyrB* mutant (Figure 4). On SBP, the expression of the arabinoxylan-specific genes was also strongly upregulated in all three Δ*larA*Δ*xyrA*Δ*xyrB*, Δ*ladA*Δ*xdhA*Δ*sdhA* and Δ*xkiA* mutants after 8 h compared to the reference strain (Figure 5B and Supplementary Table 2). However, after 24 h of incubation on both substrates, the expression of the arabinoxylan-specific genes in the Δ*xkiA* mutant was reduced at similar levels to the reference strain (Figure 5 and Supplementary Table 2), as also earlier observed for the PCP genes (Figures 3A,B).

Although cellulose is a very abundant component of both WB and SBP, the expression of the cellulose-specific genes was not significantly upregulated on WB in the mutants compared to the reference strain (Figure 5A and Supplementary Table 2). On SBP, significantly higher expression of the cellulose-specific sub-group was only observed in the Δ*larA*Δ*xyrA*Δ*xyrB* mutant (Figure 5B and Supplementary Table 2). In this mutant, increased expression of the xyloglucan-specific sub-group genes was also observed on SBP (Figure 5B). This upregulation of the cellulose-specific and xyloglucan−specific genes in the Δ*larA*Δ*xyrA*Δ*xyrB* mutant could contribute in its ability to grow on SBP (Figure 1B).

Finally, the sub-group of CAZymes with broad specificity, consisting of enzymes that act on various polysaccharides such as α−arabinofuranosidase (ABF), feruloyl esterase (FAE), β−1,4−galactosidase (LAC), β−1,4−glucosidase (BGL) and lytic polysaccharide monooxygenase (LPMO), was also significantly affected. These activities are necessary for complete depolymerization of cellulose, arabinoxylan and xyloglucan which are present in WB and the expression of some of them has been shown to be under the control of XlnR. In the conditions where the expression of the arabinoxylan-specific enzymes was increased compared to the reference strain, the expression of CAZymes with broad specificity seems to follow the same pattern (Figure 5 and Supplementary Table 2).

### The Exo-Proteome Confirmed the Large Impact of the PCP Mutants on Lignocellulolytic Enzyme Production

**FIGURE 6:**
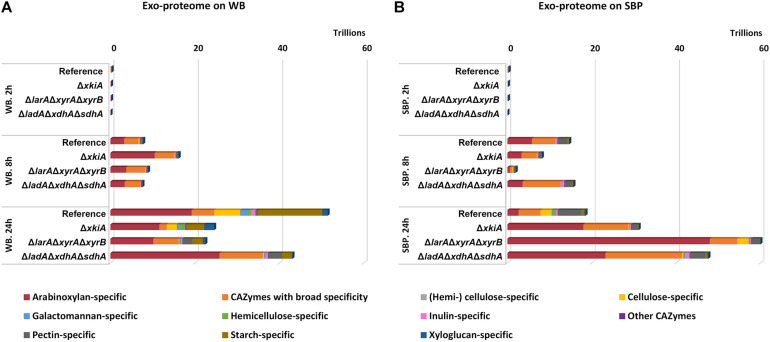
CAZy protein abundance (protein intensity values) in the secretome of the *A. niger* reference strain (N593 Δ*kusA*) and the PCP deletion mutants grown on **(A)** wheat bran (WB) and **(B)** sugar beet pulp (SBP) for 2, 8, and 24 h.

The exo-proteome of the *A. niger* reference strain and the studied PCP mutants grown on WB and SBP was also analyzed to explain their phenotypic differences on these biomass materials among the studied strains. A complete list of CAZymes secreted by the reference strain, and the Δ*larA*Δ*xyrA*Δ*xyrB*, Δ*ladA*Δ*xdhA*Δ*sdhA* and Δ*xkiA* mutants is presented in Supplementary Table 3. All experimentally identified CAZymes were qualified (number of detected CAZymes) and quantified (protein abundance). In general, a delayed response of the extracellular proteome (Figure 6) compared to the transcriptome (Figure 5) was observed. On both substrates, the strongest representation of detected CAZymes in relation to the expressed CAZy genes, as well as the highest total protein abundance were measured in the culture supernatant after 24 h of incubation (Supplementary Table 3). Additionally, differences between the transcriptomic and proteomic responses were also observed, possibly related to factors such as temporal differences, membrane binding and/or stability of the produced proteins.

On WB, the total CAZyme abundance was higher in the reference strain compared to the mutants (Figure 6), which could partly explain the reduced phenotype of the mutants on this substrate compared to the reference strain (Figure 1B). Although no significant differences were observed in the arabinoxylan-specific sub-group among the different studied stains, high variability was detected in the secreted CAZymes involved in degradation of the polysaccharides cellulose, pectin and starch (Figure 6A). On WB, the cellulose-specific CAZymes were significantly reduced in all mutants compared to the reference strain. The least affected mutant regarding the abundance of this CAZy sub-group was Δ*xkiA*, although it was still significantly reduced compared to the reference strain. This could partly explain the fact that Δ*xkiA* was the only mutant that could still grow well on WB (Figure 1B). In the other two mutants, Δ*larA*Δ*xyrA*Δ*xyrB* and Δ*ladA*Δ*xdhA*Δ*sdhA*, which were both nearly unable to utilize WB for growth, the levels of the cellulose-specific CAZymes in the secretome was dramatically reduced compared to the reference (Figure 6A and Supplementary Table 3), while the abundance of the pectin-specific CAZymes was significantly increased compared to Δ*xkiA* and the reference strain. However, the higher abundance of the pectin-specific subgroup in both mutants does not seem to be able to compensate for their reduced ability to grow on WB (Figure 1B). Finally, the secretion of the starch-specific CAZymes was significantly reduced in all three mutants compared to the reference strain. The most affected strains were the severely growth impaired Δ*larA*Δ*xyrA*Δ*xyrB* and Δ*ladA*Δ*xdhA*Δ*sdhA*, highlighting the importance of this polysaccharide during growth of *A. niger* on WB.

On SBP, the abundance of the arabinoxylan-specific CAZymes significantly varied between the reference strain and the mutants (Figure 6B). The most pronounced difference was detected in Δ*larA*Δ*xyrA*Δ*xyrB*, showing the highest production of this CAZy subgroup compared to the reference strain and Δ*ladA*Δ*xdhA*Δ*sdhA* and Δ*xkiA*. Similar to the transcriptome response (Figure 5B), the cellulose-specific CAZy sub-group in Δ*larA*Δ*xyrA*Δ*xyrB* was secreted at similar levels to the reference strain (Figure 6 and Supplementary Table 3), both showing the same growth pattern on this biomass substrate (Figure 1B). In Δ*ladA*Δ*xdhA*Δ*sdhA* and Δ*xkiA*, the abundance of the cellulose degrading CAZymes was significantly reduced compared to the reference strain. Finally, the xyloglucan-specific CAZy sub-group, which was also strongly upregulated in Δ*larA*Δ*xyrA*Δ*xyrB* grown on SBP (Figure 5B), was also secreted in significantly higher levels in Δ*larA*Δ*xyrA*Δ*xyrB*, compared to the other strains (Figure 6B).

## Discussion

In this study, the transcriptome, metabolome and proteome response of three pentose catabolic mutant strains of *A. niger*, Δ*larA*Δ*xyrA*Δ*xyrB*, Δ*ladA*Δ*xdhA*Δ*sdhA* and Δ*xkiA*, grown on two plant biomass substrates, WB and SBP, was evaluated. Both substrates contain considerable amounts of L-arabinose and D-xylose (Figure 1C), making them highly suitable for the analysis of this pathway. All three combinatorial mutations have been previously shown to block pentose catabolism at different steps of the pathway, resulting in abolishment of growth of all three mutants on L-arabinose and D-xylose, as well as in accumulation of different pathway intermediates (Chroumpi et al., 2021). However, our results reveal that despite the absolute impact of these PCP mutations during growth on pure pentose sugars, they are not as critical for growth of *A. niger* on more complex biomass substrates, such as WB and SBP.

In the Δ*xkiA* mutant, which was able to grow on both WB and SBP (Figure 1B), a strong reduction in expression of the PCP and the arabinoxylan-active CAZy genes was observed after 24 h on both substrates (Figures 3A,B, 5). Both the PCP and the arabinoxylan-specific CAZy genes have been previously shown to be under the control of the transcriptional regulators AraR and XlnR, which are specifically induced in the presence of the pentose sugars and their polyols (van Peij et al., 1998a; Battaglia et al., 2011b). Our hypothesis is that their reduced expression is due to the reduction of the arabinose, xylose and xylitol concentrations observed on both WB and SBP after 24 h in this strain, in combination with the presence of high glucose levels (Figure 4). Similar suggestions have been also made in a previous study (de Vries et al., 1999b), where the expression of the arabinoxylan-specific genes *xlnB*, *xlnD*, *aguA* and *faeA* was drastically decreased at lower concentrations of D-xylose in the presence of D-glucose, due to activation of the carbon catabolite repressor CreA. Considering that Δ*xkiA* was unable to grown on L-arabinose and D-xylose, this suggest that during growth on plant biomass a possible by-pass mechanism is induced, resulting in efficient re-routing of the metabolism of Δ*xkiA* and rescue of its growth.

**FIGURE 7:**
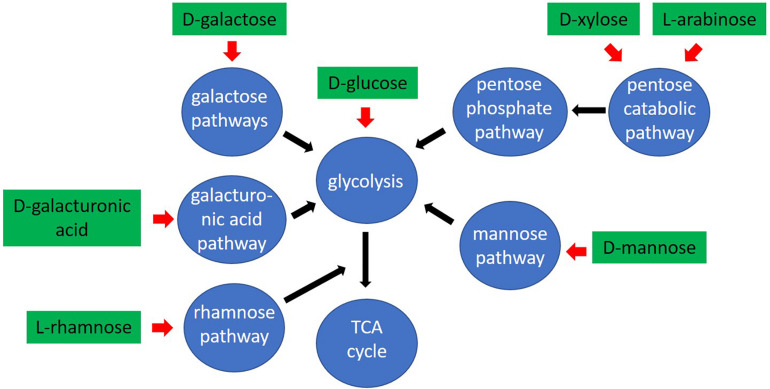
Schematic overview of the pathways used by *Aspergillus niger* to convert plant biomass derived monosaccharides.

The depletion of the pentose sugars in the Δ*xkiA* mutant, especially on WB, could be explained by the presence of enzymes with some kinase activity on D-xylulose, of which the corresponding genes are induced under these conditions. Blocking the pathway at earlier steps, as in the Δ*larA*Δ*xyrA*Δ*xyrB* and Δ*ladA*Δ*xdhA*Δ*sdhA* mutants does not result in similar rescued growth on WB (Figure 1B), suggesting that it is D-xylulose for which an alternative enzyme or pathway is induced. However, no putative enzymes, classified in the same Pfam family or showing relatively close homology to *A. niger* D-xylulokinase, were identified as likely candidates in our analysis. Alternatively, the conversion of D-xylulose into another compound that can be further metabolized through alternative carbon catabolic pathways, and not be phosphorylated, should be also considered. The better growth of the Δ*xkiA* mutant on SBP (Figure 1B), compared to WB, could be explained by the additional utilization of alternative carbon sources available for growth on this substrate (Figure 1C). SBP contains a significant amount of pectin, that next to L-arabinose also contains significant amounts of D-galacturonic acid and L-rhamnose. These sugars are converted through the GACP (Martens-Uzunova and Schaap, 2008; Alazi et al., 2017) and the RCP (Khosravi et al., 2017; Chroumpi et al., 2020; Figure 7). In Δ*xkiA*, high expression of the GACP and RCP genes were observed after 24 h (Figures 3D,F), while the accumulated D-galacturonic acid was significantly reduced after 24 h of growth on SBP (Figure 4). Higher expression of the pectin-specific CAZymes was also observed in this mutant on SBP, compared to the reference strain (Figure 5), supporting our conclusion that the presence of pectin can largely rescue growth on SBP.

The Δ*larA*Δ*xyrA*Δ*xyrB* mutant, which blocks the first step of pentose conversion (Chroumpi et al., 2021), was nearly unable to grow on WB, but only a small growth reduction was observed on SBP, compared to the reference strain (Figure 1B). As expected, accumulation of both pentoses were observed on both biomass substrates (Figure 4), while the expression of the PCP and arabinoxylan-active CAZy genes remained at high levels after 24 h (Figures 3A,B, 5). The lower expression levels of the PCP genes on SBP, compared to WB, could be explained by the different composition of these biomass substrates in pentoses (Figure 1C). Additionally, the significantly higher expression of the arabinoxylan-active CAZy genes in this mutant (Figure 5), compared to the Δ*xkiA* and Δ*ladA*Δ*xdhA*Δ*sdhA* mutants, shows that the pentose sugars and no other PCP intermediates are the main inducers of this CAZy sub-group.

In the Δ*larA*Δ*xyrA*Δ*xyrB* mutant, re-routing of sugar catabolism toward utilization of D-galacturonic acid as a substrate was also proposed, since the expression of the GACP genes (Figure 3D) was slightly increased after 24 h on SBP (Figure 4). However, low accumulation of D-galacturonic acid was observed after 24 h on SBP (Figure 4), showing reduced ability of the Δ*larA*Δ*xyrA*Δ*xyrB* mutant to metabolize D-galacturonic acid. Reduced growth and slower consumption rates of D-galacturonic acid were also previously reported for the *A. niger* Δ*larA* mutant grown on the pure sugar (Alazi et al., 2017). In their study, it was proposed that other partially redundant enzymes may also contribute in the conversion of 2-keto-3-deoxy- L-galactonate to pyruvate and L-glyceraldehyde. Deletion of the *lkaA* gene, involved in the last step of the L-rhamnose catabolism in *A. niger* (Chroumpi et al., 2020), in the Δ*gaaD* background, resulted in slightly reduced growth compared to the single Δ*gaaD* mutant (Chroumpi et al., 2021). However, growth of the double Δ*gaaD*Δ*lkaA* was not abolished on D-galacturonic acid, showing that also other enzymes are involved in this step.

The expression of the RCP genes was significantly upregulated in the Δ*larA*Δ*xyrA*Δ*xyrB* mutant, compared to the reference strain after 24 h of growth on SBP. The delayed response of the RCP genes on SBP, compared to the previously shown PCP and GACP genes, could be simply attributed to the sequential manner in which *A. niger* consumes sugars in liquid cultures (Mäkelä et al., 2018). L-rhamnose has been shown to be the least preferred carbon source between them, and as a result, the upregulation of the genes involved in its catabolism was significantly delayed compared to the other sugars (Figure 3F). Several other genes, encoding enzymes that are classified in the same Pfam family or show relatively close homology to *A. niger* pentose reductases and polyol dehydrogenases, were also found to be upregulated on SBP in the Δ*larA*Δ*xyrA*Δ*xyrB* mutant, compared to the reference strain (Supplementary Table 4). It is possible that apart from the utilization of other available sugars in SBP to support growth, these enzymes could be also involved in the rescue phenotype of this mutant on SBP, by re-routing the PCP metabolism of the Δ*larA*Δ*xyrA*Δ*xyrB* mutant. The expression of these genes was not particularly affected in the non-growing Δ*ladA*Δ*xdhA*Δ*sdhA* mutant on SBP, further supporting our theory.

In contrast to the Δ*larA*Δ*xyrA*Δ*xyrB* mutant, the growth of the Δ*ladA*Δ*xdhA*Δ*sdhA* mutant on SBP was severely impaired (Figure 1B). The Δ*ladA*Δ*xdhA*Δ*sdhA* mutant lacks *sdhA*, a gene that has been previously shown to be part of both the PCP (Chroumpi et al., 2021) and the oxido-reductive D-galactose pathway of *A. niger* (Koivistoinen et al., 2012). Thus, the growth impairment of Δ*ladA*Δ*xdhA*Δ*sdhA* on SBP could be attributed to the inability of this mutant in using both pentose sugars and D-galactose for growth. However, although this mutant was not able to grow on D-sorbitol, its growth was only slightly reduced on D-galactose compared to the reference strain (data not shown). This shows that on pure D-galactose, the D-galactose catabolism in the Δ*ladA*Δ*xdhA*Δ*sdhA* mutant can follow an alternative route than the oxidoreductive D-galactose catabolic pathway. Although, the presence of all *A. niger* genes/enzymes involved in the Leloir and the oxido-reductive D-galactose catabolic pathways have been previously shown, the relative contribution of these two pathways during growth on D-galactose or D-galactose containing complex carbohydrates is not known. It could be that under the tested conditions the oxido-reductive pathway is mainly active and as a result catabolism of D-galactose in the Δ*ladA*Δ*xdhA*Δ*sdhA* mutant is blocked, severely affecting its growth of on SBP. Finally, the potential involvement of the deleted dehydrogenase encoding genes also in other pathway steps of *A. niger* sugar catabolism or a possible severe intracellular redox imbalance effect, caused by the simultaneous deletion of these enzymes involved in NADH regeneration reactions (Witteveen et al., 1989; Koivistoinen et al., 2012), can also not be excluded. Because of the broad cellular and system functions of NAD^+^-dependent enzymes, such an imbalance in the intracellular NAD^+^/NADH ratio could alter cellular homeostasis by affecting enzymes that are involved in metabolism, regulation of gene expression, DNA repair, intracellular trafficking, aging, and cell death.

Interestingly, accumulation of D-galacturonic acid and glucose was also observed at the later time points on SBP in this mutant, suggesting that neither of these sugars could be used as alternative carbon sources. This was surprising as the Δ*ladA*Δ*xdhA*Δ*sdhA* mutant was not hypothesized to affect the D-galacturonic acid catabolism.

It was previously shown that blocking the direct entry of hexoses to the glycolytic pathway by deletion of the *hxkA* and *glkA* genes activates alternative metabolic conversion of these sugars in *Aspergillus nidulans* during growth on WB, but also upregulates conversion of other sugars, such as pentoses (Khosravi et al., 2018). However, in our study, although both substrates are rich in cellulose, this does not seem to significantly compensate for their reduced ability to use pentoses. In all the PCP mutants, the expression of the glycolytic genes on both substrates was unaffected, compared to the reference strain (data not shown). Additionally, neither the genes encoding cellulose-active enzymes were significantly upregulated in most of the PCP mutants (Figure 5) nor extracellular detection of these enzymes was observed in the proteomic data (Figure 6). Only in the Δ*larA*Δ*xyrA*Δ*xyrB* mutant, the cellulase-specific CAZy genes were upregulated on SBP (Figure 5B) and earlier reduction of the accumulated glucose levels was observed (Figure 4), which may have also contributed to its rescued growth on this substrate. Improved cellulase production by *A. niger* has been previously achieved by deleting the *noxR* gene, encoding the regulatory subunit of the NADPH oxidase complex (Patyshakuliyeva et al., 2016). A combination of the PCP and *noxR* gene deletions might help to improve cellulase utilization of these strains and as a result the growth of the PCP mutants on these biomass substrates.

Our results also suggests that an adaptation to other components of the substrates may cause a general growth arrest. This was in particular true for SBP as at 8 h an overall repression of genes involved in most of the studied GO terms was observed for all three mutants. This could be an indication that at that later time point the mutants’ metabolism is paused, due to the fact that the most available carbon sources L-arabinose and D-xylose cannot be used for growth, before they redirect their metabolism and adapt in the new situation.

To conclude, our results demonstrate that despite the significant impact of these catabolic gene deletions during growth on pure pentose sugars, they are not as critical for growth of *A. niger* on more complex biomass substrates. It seems that the high sugar heterogeneity of these substrates in combination with the high complexity and adaptability of the fungal sugar metabolism allow for activation of alternative strategies to support growth. Production of additional enzymes that have side-activity on the PCP sugars, and therefore contribute to the conversion of D-xylose or L-arabinose, or re-routing of sugar catabolism toward utilization of other available plant derived monosaccharides apart from pentoses were shown to be involved. This advanced understanding of central metabolic pathways is critical when applying metabolic engineering strategies in biotechnology. The use of low-cost lignocellulosic biomass materials as feedstocks combined with metabolic engineering could facilitate efficient utilization of the raw materials, but high production rates and high growth rates are required to attain economically feasible biotechnological processes. The effects of the mutations on CAZy genes were less clear, which could be due to the fact that many of these genes are affected by multiple regulators in *A. niger* (Gruben et al., 2017).

## Data Availability Statement

The datasets presented in this study can be found in online repositories. The names of the repository/repositories and accession number(s) can be found below: https:// massive.ucsd.edu/, MSV000086614; https://www.ncbi.nlm.nih.gov/genbank/, GSE162901.

## Author Contributions

TC performed the experiments, analyzed the data, and wrote the manuscript. MP contributed to the bioinformatics analysis and data visualization. LM, HM, CN, CH, VP, NT, CC, GO, and SB performed the RNA sequencing and metabolomic and proteomic analyses. RV and TC designed the study. RV and MM critically revised the manuscript. All authors contributed to the article and approved the submitted version.

## Conflict of Interest

The authors declare that the research was conducted in the absence of any commercial or financial relationships that could be construed as a potential conflict of interest.

## Footnotes

1 https://www.bioinformatics.babraham.ac.uk/projects/fastqc/

2 http://bowtiebio.sourceforge.net/bowtie2/index.shtml

3 https://genome.jgi.doe.gov/Aspni_NRRL3_1/Aspni_NRRL3_1.home.html

4 http://www.aspgd.org/

5 https://github.com/PNNL-Comp-Mass-Spec/MASIC/releases

6 https://github.com/PNNL-Comp-Mass-Spec/InfernoRDN/releases

## Supplementary Material

Supplementary Table 1 Expression levels (FPKM) of catabolic genes during growth on wheat bran (WB) and sugar beet pulp (SBP) in the *A. niger* reference strain (N593 ΔkusA) and the PCP deletion mutants.

Supplementary Table 2 Expression levels (FPKM) of genes encoding CAZymes during growth on wheat bran (WB) and sugar beet pulp (SBP) in the *A. niger* reference strain (N593 ΔkusA) and the PCP deletion mutants.

Supplementary Table 3 Comparison between the transcriptome and exo-proteome analysis of the *A. niger* reference strain (N593 ΔkusA) and the PCP deletion mutants grown on wheat bran (WB) and sugar beet pulp (SBP) for 2, 8, and 24 h. Transcripts were considered differentially expressed if the DESeq2 fold change was >2 (red font) or <0.5 (green font) and Padj <0.01. Similarly, for an identified protein to be considered differentially produced, the requirement was a fold change of the mean intensity values of >2 (highlighted in red) or <0.5 (highlighted in green) and Padj <0.05. Note that where an intensity value was not detected for a protein in a sample, a zero value was used. “Inf” values shown in the fold change columns are caused by zero values in the mutant. All values are averages of triplicates. The fold change is the difference between the reference strain and the deletion mutants.

Supplementary Table 4 Expression levels (FPKM) of genes encoding enzymes that are classified in the same Pfam family or show relatively close homology to *A. niger* pentose reductases and polyol dehydrogenases, during growth on (A) wheat bran (WB) and (B) sugar beet pulp (SBP) in the *A. niger* reference strain (N593 ΔkusA) and the PCP deletion mutants. Transcripts were considered differentially expressed if the DESeq2 fold change was > 2 (highlighted in orange) or <0.5 (highlighted in green) and Padj <0.01. Transcripts with FPKM ≤50 were considered lowly (i.e., not substantially) expressed and highlighted in red font. The values are averages of triplicates. The fold change is the difference between the reference strain and the deletion mutants.
